# Modeling and Characterization of Scaling Factor of Flexible Spiral Coils for Wirelessly Powered Wearable Sensors

**DOI:** 10.3390/s20082282

**Published:** 2020-04-17

**Authors:** Dipon K. Biswas, Melissa Sinclair, Tien Le, Salvatore Andrea Pullano, Antonino S. Fiorillo, Ifana Mahbub

**Affiliations:** 1 Department of Electrical Engineering, University of North Texas, Denton, TX 76203, USA; MelissaSinclair@my.unt.edu (M.S.); TienLe@my.unt.edu (T.L.); Ifana.Mahbub@unt.edu (I.M.); 2 Department of Health Sciences, University of Magna Græcia, 88100 Catanzaro, Italy; pullano@unicz.it (S.A.P.); nino@unicz.it (A.S.F.)

**Keywords:** inductive power transmission, flexible AC transmission systems, wireless power transmission, wearable sensors, energy efficiency

## Abstract

Wearable sensors are a topic of interest in medical healthcare monitoring due to their compact size and portability. However, providing power to the wearable sensors for continuous health monitoring applications is a great challenge. As the batteries are bulky and require frequent charging, the integration of the wireless power transfer (WPT) module into wearable and implantable sensors is a popular alternative. The flexible sensors benefit by being wirelessly powered, as it not only expands an individual’s range of motion, but also reduces the overall size and the energy needs. This paper presents the design, modeling, and experimental characterization of flexible square-shaped spiral coils with different scaling factors for WPT systems. The effects of coil scaling factor on inductance, capacitance, resistance, and the quality factor (*Q*-factor) are modeled, simulated, and experimentally validated for the case of flexible planar coils. The proposed analytical modeling is helpful to estimate the coil parameters without using the time-consuming Finite Element Method (FEM) simulation. The analytical modeling is presented in terms of the scaling factor to find the best-optimized coil dimensions with the maximum *Q*-factor. This paper also presents the effect of skin contact with the flexible coil in terms of the power transfer efficiency (PTE) to validate the suitability as a wearable sensor. The measurement results at 405 MHz show that when in contact with the skin, the 20 mm× 20 mm receiver (RX) coil achieves a 42% efficiency through the air media for a 10 mm distance between the transmitter (TX) and RX coils.

## 1. Introduction

**Figure 1:**
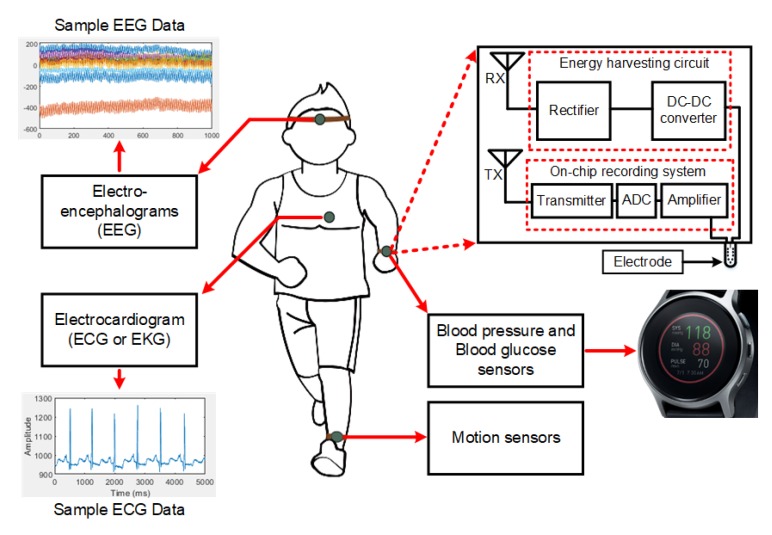
Wirelessly powered wearable sensor applications.

Wearable body sensor network (WBSN) interconnects a network of heterogeneous sensors, which, as outlined in Figure 1, is becoming even more popular in long-term health monitoring and diagnostics [1,2,3]. Continuous glucose monitoring, which helps to control diseases like diabetes, is one of the most common examples of health monitoring applications [4]. The use of wearable potentiometric sensors to analyze the biofluids such as saliva, tears, and urine open up the new direction of using sensors for subcutaneous glucose monitoring [5]. Wearable sensors are typically patched to the skin or embedded into the clothes, requiring high flexibility and conformability [6]. Polyethylene naphthalate (PEN) [7], polyimide [8], poly-di-methyl-siloxane (PDMS) [9], parylene [10], and Kapton [11] are just a few examples of flexible substrates that have been investigated as wearable sensor platforms. Besides the flexibility and conformability, efficient power delivery to the sensors is also an important aspect that needs to be taken into consideration in wearable sensor system design. Batteries are traditionally used to power up WBSN, even though they require frequent charging and occasional replacements. To avoid the operational complexity of replacing the batteries, optical fibers are used to provide power to the batteries using light [12]. However, this technique limits the natural movement and the working range of the system [13]. Alternatively, in prior works, sensors are designed to be powered by a near-field inductive-coupling wireless power transfer (IWPT) scheme [14,15,16]. IWPT uses two or more coils that are separated by a medium (e.g., air or tissue layers). An oscillating current generates an electromagnetic field that is emitted from the primary coil and induces an electric current in the secondary coil. The IWPT performance mainly depends on the substrate materials, coil geometry, and the distance between the coupled coils [17,18]. Various applications require various dimensions of the coil, and for rapid prototyping, it is highly important to model the coils in terms of scaling factors. There is no prior work that has focused on this aspect of coil modeling, especially in the context of wearable, flexible sensor applications.

Hereafter, the design and characterization of the secondary receiver (RX) coil for an IWPT is investigated in this paper, analyzing the effects of coil scaling on parameters such as *Q*-factor and PTE. This paper also investigates how the coil scaling changes the parameters of the lumped component-based modeling (e.g., resistance, inductance, and parasitic capacitance) that eventually affect the coil’s properties: self-resonating frequency, *Q*-factor, PTE, etc. The objective of this paper is to establish a mathematical model to identify the optimum scaling factor that would result in the best-optimized RX coil design in terms of the *Q*-factor and the PTE. The mathematical model also takes into account the effect of skin contact with the substrate and how that affects the PTE for a particular transmitter–receiver (TX–RX) pair. For a quick and rapid prototyping, having a model with respect to the scaling factor is better. The rest of the paper is organized as follows. Section 2 presents the analysis and the modeling of the spiral coil in terms of the scaling factor. Section 3 presents the implemented IWPT scheme and the fabrication procedure of the flexible spiral coils on Kapton substrate. In Section 4, the experimental results depicting the correlation between the scaling factor and the *Q*-factor, and the lumped element modeling are presented. This is then followed by the conclusion in Section 5.

## 2. Analytical Spiral Coil Modeling

**Figure 2:**
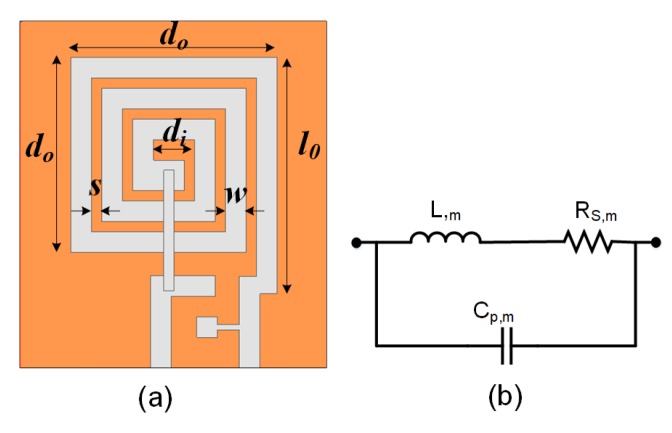
(**a**) Layout of the designed spiral coil and the related representative dimensions, (**b**) electrical model of the receiver (RX) coil.

The designed coil has five significant parameters that are highlighted in Figure 2a: outer diameter, $d_{o}$; inner diameter, $d_{i}$; initial trace length, $l_{0}$; conductor trace width, *w*; and gap between traces, *s*. The RX design is optimized based on the optimization algorithm presented in our prior work [19], which takes into account the initial parameters and design constraints, such as the minimum and maximum value range for $d_{o}$, *w*, and *s*, to find out the best possible design in terms of the maximum PTE performance. In this work, the RX spiral coil is electrically modeled in terms of the resistance $R_{S,m}$, inductance $L_{,m}$, and parasitic capacitance $C_{P,m}$, as shown in Figure 2b. In this paper, the values of $R_{S,m}$, $L_{,m}$, $C_{P,m}$, and $f_{SRF,m}$ for *m* times scaled coil (where all the parameters in Figure 2a are scaled *m* times) are calculated based on the analytical modeling, which is later validated through the simulation and measurement results.

For an *m*-scaled coil, the total length of the conductor trace is $l_{c,m}$, which can be modeled as

(1) $$l_{c,m}=m\left(l_{0}+\left(4n-1\right)d_{0}-4n^{2}w-{\left(2n-1\right)}^{2}s\right)$$

Here, *n* denotes the number of turns of the coil. When *m* = 1, the total length of the conductor trace of the base coil is determined and denoted as $l_{c,1}$. Thus, a linear relationship between the $l_{c,m}$ and $l_{c,1}$ can be derived as

(2) $$l_{c,m}=m\cdot l_{c,1}$$

The total length of the coil $l_{c,m}$ is used to determine the DC resistance, $R_{dc,m}$, of the coil as

(3) $$R_{dc,m}=\rho_{c}\frac{l_{c,m}}{m\cdot w\cdot t_{c}}$$

where $\rho_{c}$ is the resistivity of the trace of the coil and $t_{c}$ is the thickness of the metal trace. The value of $R_{dc,m}$ is thus independent of the value of *m*, as shown in Equation (4).

(4) $$R_{dc,m}=R_{dc,1}$$

Due to the increase of the coil resistance at high frequency, the skin effect is also taken into consideration in the modeling. Thus, the total resistance, $R_{S,m}$ can be evaluated as [20]:

(5) $$R_{S,m}=R_{dc,m}\cdot \frac{t_{c}}{\delta (1-e^{\frac{t_{c}}{\delta }})}$$

(6) $$\mathrm{where}\;\delta =\sqrt{\frac{\rho_{c}}{\pi \mu f}},\mu =\mu_{r}\mu_{0}$$

where δ is the skin depth, $\mu_{0}$ is the permeability of the free space, and $\mu_{r}$ is the relative permeability of the trace conductor.

As $R_{dc,m}$ is independent of *m* and $t_{c}$ does not change with *m*, the value of $R_{S,m}$ is proven to be independent of the value of *m*.

(7) $$R_{S,m}=R_{S,1}$$

where $R_{s,1}$ is the total resistance when *m* = 1. Due to the epoxy used for soldering components, additional resistance needs to be added with the total resistance, which is empirically found and discussed in the later section.

The inductance of the coil with a corresponding scaling factor *m* is denoted as $L_{,m}$, which can be calculated from the base coil inductance [21]. With the consideration of the scaling factor *m*, the value of $L_{,m}$ can be evaluated as

(8) $$L_{,m}=\frac{1.27\cdot \mu_{0}n^{2}d_{avg,m}}{2}\left[ln\left(\frac{2.07}{\varphi_{m}}\right)+0.18\varphi_{m}+0.13\varphi_{m}^{2}\right]$$

where $\varphi_{m}$ is the fill factor of the coil and $d_{avg,m}$ is the average of *m* times of the $d_{o}$ and $d_{i}$ of the base coil.

(9) $$\varphi_{m}=\frac{m\cdot (d_{0}-d_{i})}{m\cdot (d_{0}+d_{i})};\varphi_{m}=\varphi_{1}$$

(10) $$d_{avg,m}=\frac{m\cdot (d_{0}+d_{i})}{2}=m\cdot d_{avg,1}$$

where $\varphi_{1}$ is the fill factor and $d_{avg,1}$ is the average diameter of the coils when *m* = 1.

(11) $$\mathrm{Thus},\;L_{,m}=m\cdot L_{,1}$$

Therefore, the inductance shows a linear relationship with the scaling factor. According to the authors of [20], the coil’s parasitic capacitance is analyzed through the substrate and across the air gap between the traces. $C_{P,m}$ is the parasitic capacitance of a scaled-*m* coil and can be determined as shown in Equation (12), where $\epsilon_{rs}$ and $\epsilon_{rc}$ are the relative dielectric constants of the substrate and the coating of the substrate, respectively. α and β are constants that are discussed in Equations (16) and (17), respectively.

(12) $$C_{P,m}\approx \left(\alpha \epsilon_{rc}+\beta \epsilon_{rs}\right)\epsilon_{0}\frac{t_{c}}{m\cdot s}l_{g,m}$$

As the length, $l_{g,m}$, and the spacing, *s*, increase by *m* factor and that cancels each other out, the value of $C_{P,m}$ is also independent of *m*, as shown in Equation (13).

(13) $$C_{P,m}=C_{P,1}$$

(14) $$l_{g,m}=m\left(4\left(d_{0}-w\cdot n\right)\left(n-1\right)-4sn\left(n+1\right)\right)$$

(15) $$l_{g,m}=m\cdot l_{g,1}$$

where $l_{g,m}$ is the total length of the spacing or gap length between the traces. The term α and β mentioned in Equation (12) are the attenuation and phase constants of the substrate, respectively:

(16) $$\alpha =2\pi f\sqrt{\frac{\mu \epsilon^{\prime }\epsilon_{0}}{2}}{\left(\sqrt{1+{\left(\frac{\epsilon^{\prime \prime }}{\epsilon^{\prime }}\right)}^{2}}-1\right)}^{\frac{1}{2}}$$

(17) $$\beta =2\pi f\sqrt{\frac{\mu \epsilon^{\prime }\epsilon_{0}}{2}}{\left(\sqrt{1+{\left(\frac{\epsilon^{\prime \prime }}{\epsilon^{\prime }}\right)}^{2}}+1\right)}^{\frac{1}{2}}$$

where *f* is the operating frequency, $\epsilon^{\prime }$ and $\epsilon^{\prime \prime }$ are the real and imaginary part of the permittivity of the substrate, respectively. An empirically found capacitance due to the epoxy used in fabrication is also added with the parasitic capacitance, $C_{P,m}$ which is further discussed in Section 4.

The self-resonating frequency $f_{SRF,m}$ is the frequency when capacitive reactance, $X_{C}$, and inductive reactance, $X_{L}$, are equal.

(18) $$X_{C}=\frac{1}{2\pi f_{SRF,m}C_{P,m}}$$

(19) $$X_{L}=2\pi f_{SRF,m}L_{,m}$$

By making $X_{L}$ and $X_{C}$ equal and by substituting Equations (8) and (12) in Equations (18) and (19), an Equation can be derived allowing $f_{SRF,m}$ to be calculated as

(20) $$\begin{array}{c} f_{SRF,m}=\{4\pi^{2}2\pi \epsilon_{0}\sqrt{\frac{\mu \epsilon^{\prime }\epsilon_{0}}{2}}\cdot [{\left(\sqrt{1+{\left(\frac{\epsilon^{\prime \prime }}{\epsilon^{\prime }}\right)}^{2}}-1\right)}^{\frac{1}{2}}\epsilon_{rc}\\ +{\left(\sqrt{1+{\left(\frac{\epsilon^{\prime \prime }}{\epsilon^{\prime }}\right)}^{2}}+1\right)}^{\frac{1}{2}}\epsilon_{rs}]\cdot \frac{t_{c}}{m\cdot s}l_{g,m}L_{,m}{\}}^{-\frac{1}{3}} \end{array}$$

According to the work presented by Jow et al. [20], when the resistivity $R_{S,m}$, inductance $L_{,m}$, and parasitic capacitance $C_{P,m}$ of a coil are known, the coil’s *Q*-factor can be calculated using Equation (21).

(21) $$Q=\frac{\omega L_{,m}-\omega \left(R_{S,m}^{2}+\omega^{2}L_{,m}^{2}\right)C_{P,m}}{R_{S,m}};\omega =2\pi f$$

(22) $$\mathrm{Thus},\;Q\left(m\right)=am^{2}+bm+c$$

(23) $$\mathrm{where}\;a=\frac{-\omega^{3}L_{,1}^{2}C_{P,1}}{R_{S,1}};b=\frac{\omega L_{,1}}{R_{S,1}};c=-\omega R_{S,1}C_{P,1}$$

Thus, the *Q*-factor formula can be simplified as Equation (22). For the case where the values of $d_{o}$, $d_{i}$, $l_{0}$, *w*, *s*, *n*, and $t_{c}$ are known and remain constant, the values of $L_{,1}$, $C_{P,1}$, and $R_{S,1}$ in Equation (23) will be constant. Thus, at a given frequency *f*, *Q* of an *m*-scaled coil becomes a quadratic function of *m*. As this quadratic function has a negative leading coefficient *a*, the graph of the function is a parabola curve that opens downward and has its maximum value at the vertex of the function. The horizontal coordinate of the vertex is calculated using Equation (24), allowing the maximum value of the *Q*-factor, $Q_{max}$, to be determined, as shown in Equation (25).

(24) $$m_{vertex}=\frac{-b}{2a}=\frac{1}{2\omega^{2}L_{,1}C_{P,1}}$$

(25) $$Q_{max}=Q\left(\frac{-b}{2a}\right)=\frac{1}{4\omega R_{S,1}C_{P,1}}-\omega R_{S,1}C_{P,1}$$

The PTE determines the effectiveness of the WPT system at delivering power. The PTE depends on the coupling coefficient *k* and *Q*-factor of the primary and the secondary coils. The link efficiency of the WPT system is derived as in [22] where $Q_{1}$ and $Q_{2}$ are the *Q*-factors of the primary and the secondary coils, respectively, and $Q_{L}$ is the loaded *Q*-factor of the secondary coil.

(26) $$\eta_{12}=\frac{k^{2}Q_{1}Q_{L}}{1+k^{2}Q_{1}Q_{L}}\cdot \frac{Q_{L}}{Q_{2}+Q_{L}};Q_{L}=\frac{1}{2}\sqrt{\frac{R_{L}}{R_{S,1}}}$$

where $R_{L}$ is the load resistance and $R_{S,1}$ is the total resistance of the RX coil.

The coupling coefficient, *k*, is a function of the distance between the primary and the secondary coils, *d*, and the radius of the coils [23,24].

(27) $$k=\frac{1}{1+{\left(2^{\frac{2}{3}}\cdot (\frac{d}{\sqrt{R_{pc}\cdot R_{sc}}}{)}^{2}\right)}^{\frac{3}{2}}}$$

where $R_{pc}$ and $R_{sc}$ denote the radii of the primary and the secondary coils, respectively. As the radius of the secondary coil $R_{sc}$ increases by *m*, the outer diameter $d_{0}$ also increases by *m*, and therefore the coupling coefficient, *k*, increases by $∼m^{3/2}$ according to Equation (27).

## 3. Architecture and Fabrication

### 3.1. Fabrication

**Figure 3:**
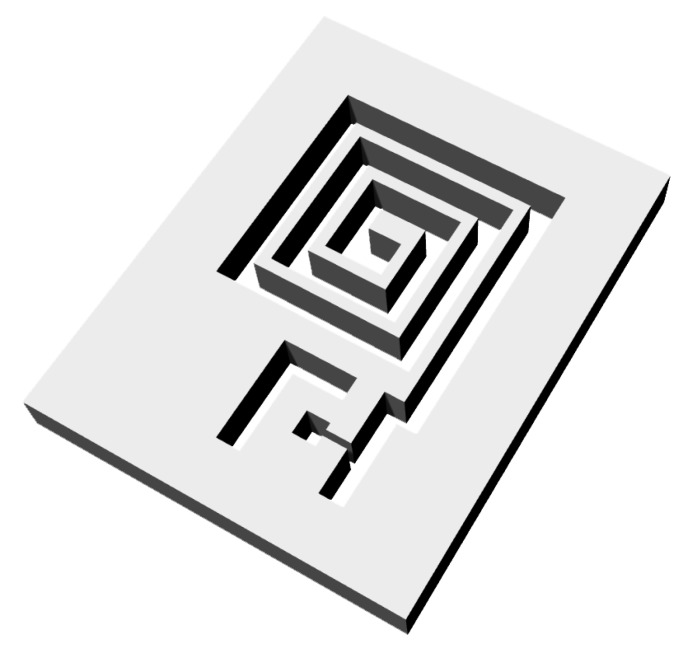
Rendering of the designed 3D-CAD stencil mask.

Kapton polyimide film of 25 μm in thickness (DuPont^TM^ Kapton^®^ Coveme 100 NH) is chosen as the flexible substrate for its physical robustness, flexibility at a wide range of temperatures (up to 400 ${}^{\circ }$C with a thermal deformation of less than 10%), and a high dielectric strength (≈177 kV/mm) [25]. The patterning of coil structures on the substrate uses a stencil mask for the conductive layer deposition. For iterative rapid prototyping, the stencil mask is 3D printed. The stencil mask is designed using a 3D-CAD software (Rhinoceros^®^, Seattle, WA) as shown in Figure 3, and is printed using Acrylonitrile Butadiene Styrene (ABS) using a 3D printer (Stratasys Uprint SE, Brooklyn, NY, USA). The trade-off of the rapid prototyping using ABS stencil mask is that the mask edge is characterized by a higher roughness with respect to a standard metal mask. Silver deposition on the substrate is realized by e-beam physical vapor deposition (Varian technologies, USA) at a pressure of 5 × 10${}^{-6}$ Torr with a deposition rate of 1 nm/s resulting in a thickness, $t_{c}$, of 0.25 μm (Table 1). The film thickness is controlled by a quartz crystal microbalance (Inficon, Switzerland). SMA connectors are bonded to the Kapton substrate using a silver-loaded conductive epoxy (MG Chemicals^®^ 8331-B). A thin copper wire is used to connect the center of the coil to the center pin of the SMA connector as well.

### 3.2. Secondary Coil

**Figure 4:**
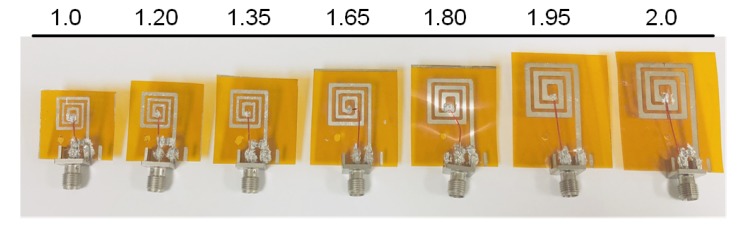
Fabricated scaled flexible coil prototypes.

By scaling the base geometry, a set of *m* coils are obtained. For this work, the values of *m* chosen for fabrication are 1.00, 1.20, 1.35, 1.65, 1.80, 1.95, and 2.00, as shown in Figure 4. Table 2 lists the actual values of these parameters of the base coil as well as the seven different *m*-scaled coils. To investigate the effects of scaling factor, *m*, on the characteristics of the fabricated planar-spiral coil, a model (*m* = 1.00) coil is designed as the base design using the optimization algorithm presented in [19]. The base coil and the *m*-scaled coils have the same number of turns, *n*, of 3; conductor thickness, $t_{c}$, of 0.25 μm; and substrate thickness, *h*, of 25 μm. The outer diameter, $d_{o}$, is set to be 10.5 mm, and the inner diameter, $d_{i}$, is 2.1 mm for the base coil.

### 3.3. Primary Coil

**Figure 5:**
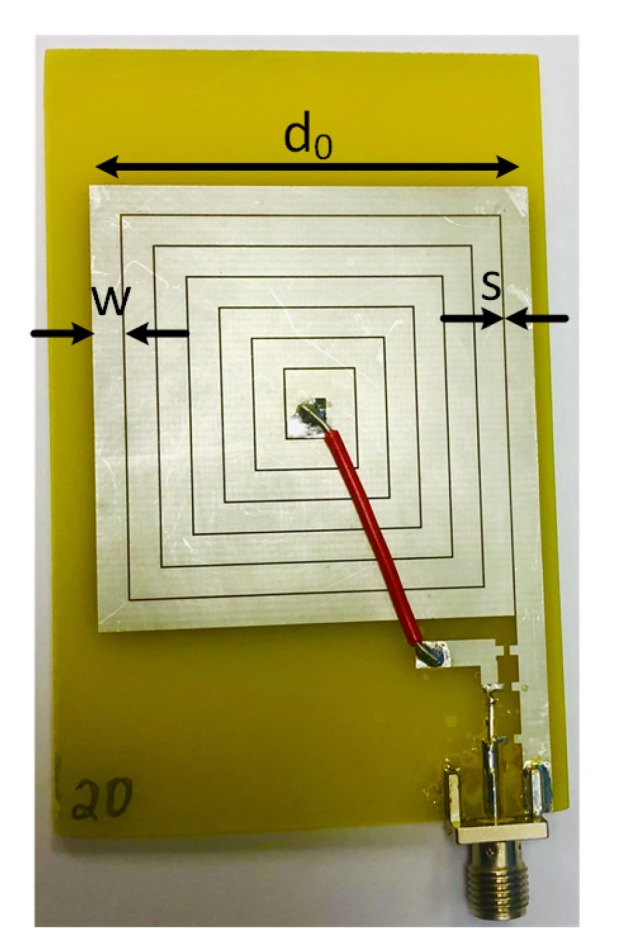
Transmitter (TX) coil fabricated on Cu-clad FR4 substrate.

To evaluate the performance of the fabricated coils as a WPT system, a planar TX coil is fabricated on the FR4 board, which is a 7-turn coil with 45 mm × 45 mm dimensions. The square-shaped spiral coil is fabricated using a copper conductor. As shown in Figure 5, the outer diameter, $d_{0}$, of the TX coil is 45 mm with the trace width, *w*, of 3 mm and trace spacing, *s*, of 0.2 mm.

## 4. Results and Discussion

**Figure 6:**
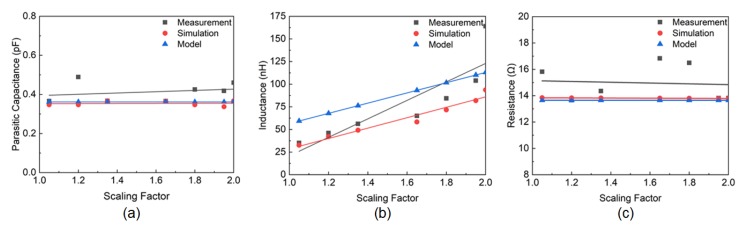
(**a**) Parasitic capacitance at self-resonant frequency with respect to the scaling factor, *m*. (**b**) Inductance with respect to the scaling factor, *m*. (**c**) Resistance with respect to the scaling factor, *m*.

The electromagnetic simulations are performed using Ansys High-Frequency Structures Simulator (HFSS) software for the proposed coil models. The simulated results are used to analyze the impedance of the coils. For the measurement of the coils, the ZVB 20 Vector Network Analyzer (Rohde & Schwarz) is used to evaluate the impedance. From the acquired real and imaginary parts of the impedance, the values of $R_{dc,m}$, $R_{S,m}$, and $L_{,m}$ are evaluated, while $C_{P,m}$ is evaluated using Equation (12). The modeling, simulation, and measurement results of $L_{,m}$, $C_{P,m}$, and $R_{S,m}$ are shown in Figure 6a–c, respectively. In Figure 6a, it is evident that the parasitic capacitance of the measured coil is slightly higher than the theoretical, and simulated models. To evaluate the accuracy of the model, the average error percentage for the simulated and the measured results is calculated using Equation (28), where *N* is the number of data points.

(28) $$Error_{avg}=\frac{\sum \frac{|Simulated/Measured-Modeled|}{Modeled}\times 100\%}{N}$$

The percentage error is calculated to be 1.9% for the simulated results, whereas the measured results show a 13.8% average error compared to the theoretical model. Though an additional capacitance of approximately 350 fF due to the epoxy required for soldering SMA connector with the antenna is incorporated in both analytical and simulated models, the added capacitance is potentially due to the uneven traces during manufacturing. The printing of the stencil mask is inherently characterized by the variations in width due to the transition from liquid to the solid phase of ABS which causes the variations in trace width.

Based on Equation (11), the inductance $L_{,m}$ shows a linear relationship with the scaling factor *m* which is also evident from Figure 6b. The measured and simulated results also show a linear relationship with respect to the *m*. However, the modeled inductance is significantly higher due to having a lower parasitic capacitance as previously discussed.

The equivalent resistance of the silver coil is shown in Figure 6c. The theoretically modeled and the simulated results show a steady constant relationship while the measured resistance has a slight decrease. The measured resistance is also found to be higher than the modeled and simulated results due to the uneven traces of the fabricated coils. The additional resistance is added due to the epoxy and the SMA connector connected to the fabricated device. From the empirical data, the resistance value is found to be ∼ 8.7 Ω, which is considered in the modeled and simulated data. According to Equation (28), approximately 0.72% and 9.3% errors in simulated and the measured results compared to the theoretical model are found for the resistance, respectively.

**Figure 7:**
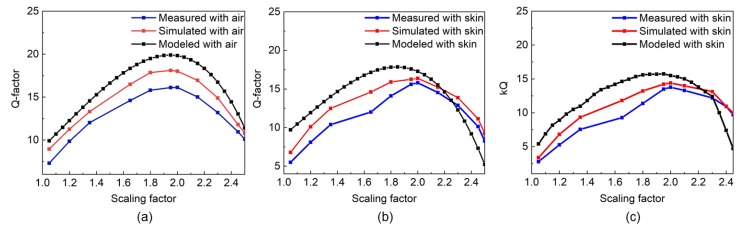
Modeled, simulated, and measured Q-factor with respect to the scaling factor, *m* (**a**) without skin contact, (**b**) with skin contact, and (**c**) kQ with respect to the scaling factor.

According to Equation (22), the *Q*-factor is a quadratic function of the scaling factor *m*. Furthermore, at a fixed frequency *f*, the maximum value of the *Q*-factor, $Q_{max}$, can be determined at the vertex of the quadratic Equation where *m* is equal to $m_{vertex}$. Therefore, at a fixed frequency, a coil needs to be scaled by a factor of $m_{vertex}$ to achieve the maximum value of the quality factor. The simulated *Q*-factor does not account for uneven trace thickness that might be the case for the fabricated coils. By using Equation (25), the modeled maximum *Q*-factor can be determined. Although some discrepancies are observed between the theoretically modeled and the measurement results due to the fringe capacitance effects, the model can still predict the behavior of the coil for different scaling ratios. As shown in Figure 7a, the modeled Q-factor shows the highest value of 20 for the scaling factor of 2.00 at 405 MHz frequency. The frequency of operation is in the Medical Implant Communication Service (MICS) band, as the flexible RX coils are meant to be used for wearable sensing applications. From Equation (24), the calculated $m_{vertex}$ value is also found to be 2.00, showing alignment with the simulation and measurement results. There are discrepancies between the measured-modeled and simulated-modeled results according to Figure 7a. Though the modeled resistance and capacitance are very close to the simulated values, the modeled inductance is slightly higher than the simulated values, which reduces the overall *Q*-factor of the simulated coil. The difference between the modeled and the measured *Q*-factor is due to the higher resistance and capacitance values that are found from the measurement results. To evaluate the performance of these flexible coils as wearable sensors, a set of measurements are also performed with the coils in contact with the skin. In the modeling, the only variable factor is the relative dielectric constant of the substrate, $\epsilon_{rs}$, in Equation (12), which is changed due to the addition of the dielectric constant of the skin and other tissue layers underneath the substrate. If the dielectric constant of the skin layer is $\epsilon_{skin}$ and the thickness of the skin layer is represented as $d_{skin}$, the effective dielectric constant due to the skin layer and the substrate can be represented as Equation (29) [26].

(29) $$\epsilon_{eff}=\frac{d_{rs}+d_{skin}}{\frac{d_{rs}}{\epsilon_{rs}}+\frac{d_{skin}}{\epsilon_{skin}}}$$

Here, $d_{rs}$ represents the thickness of the substrate layer. As the overall dielectric constant increases due to the contact with the skin, the maximum *Q*-factor for different scaling factors is also changed according to Equation (25). As shown in Figure 7b, the theoretically modeled *Q*-factor with the skin contact is reduced compared to the *Q*-factor without any skin contact. The simulated and the measured results also follow the same pattern as the theoretical model. Due to the skin contact, the modeled, simulated, and measured *Q*-factors of the *m*-scaled coils are decreased by approximately 14% compared to the *Q*-factor values without the skin contact. However, the maximum *Q*-factor still shows the maximum value around $m_{vertex}$∼ 2.00. To better understand the effect of the scaling factor, the product of the coupling coefficient, *k*, and *Q*-factor is plotted with respect to the scaling factor as shown in Figure 7c. With the increase of the scaling factor of the RX coil, the value of *k* increases for a fixed TX coil and for a fixed distance between the TX and RX coils. The fabricated TX coil is used to find the value of *k* and the distance between the TX and RX coils is kept to be 10 mm. As a result, the product, kQ, follows a similar trend as the *Q*-factor plot, which shows the maximum kQ value at ∼ 2.00 scaling factor.

**Figure 8:**
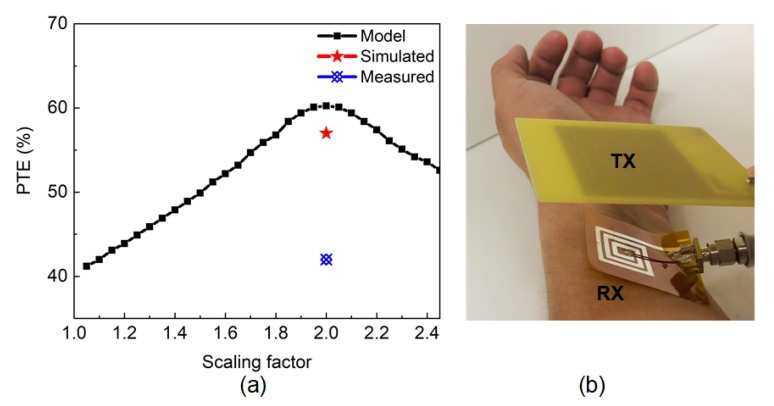
(**a**) Modeled, simulated, and measured PTE with the skin contact. (**b**) Test set-up for the measurement of PTE with the skin contact.

The PTE is theoretically modeled for the desired resonating frequency, 405 MHz, for all the scaling factors, which is shown in Figure 8a. As the fabricated Kapton-based coil is intended to be used as a wearable sensor, the PTE is also modeled, simulated, and measured when the RX coil is in contact with the skin. The modeled PTE shows an increasing trend over the scaling factor as shown in Figure 8a. With the increase in the scaling factor, the coupling coefficient increases. In this work, the distance between the TX and RX coils are kept as 10 mm. As a result, the coupling coefficient is only dependent on the RX size which is a linear function of the scaling factor. Thus, the PTE also shows an increase with the increase of the scaling factor. The RX coil with the scaling factor of 2.00 is used in the simulation and measurement of the PTE due to its high *Q*-factor values. The simulated and measured PTE, η are calculated from the transmitted power, $P_{tx}$ and received power, $P_{rx}$ as shown in Equation (30).

(30) $$\eta =\frac{P_{tx}}{P_{rx}}\times 100\%$$

To resonate the RX coil at the 405 MHz operating frequency, 1 pF capacitor is added in parallel with the coil. The *Q*-factor of the fabricated TX coil is 134, and a parallel capacitor of 3.3 pF is used to resonate the TX to 405 MHz. The test set-up for PTE measurement with skin contact is shown in Figure 8b. For a transmitted power of 19 dBm, the system can achieve approximately 42% measured PTE having the skin in contact when the distance between the TX and RX coil is 10 mm. On the other hand, in simulation, 57% PTE is achieved for 2.00 scaled RX coil. The discrepancies between the PTE results are due to the bending effect during the measurements which is not taken into consideration in the simulation or mathematical modeling. The theoretical modeling of the bending effect of the flexible substrate is planned to be considered in the future works to improve the model. The performance of the proposed WPT system is also compared with the state-of-the-art systems in Table 3. As the wearable WPT systems can be used to provide power to the electrodes or other sensors, PTE of the WPT system is very important. The measured PTE of the proposed flexible WPT system is 42% which is much higher than the other works and thus, the system is able to provide more power to the load circuitry. The proposed modeling provides a systematic way to choose the best-optimized architecture for the wearable sensor applications. On the contrary, prior works did not follow any modeling Equation to find the optimized design.

## 5. Conclusions

For implementing an IWPT system, the efficiency of the WPT system is determined by the PTE. The coil dimensions affect the coupling coefficient between the primary and the secondary coils, which affects the value of the PTE. Therefore, the IWPT can be improved by analyzing how proportional scaling factors impact the self-resonant frequency, inductance, parasitic capacitance, *Q*-factor, and the resistivity of the receiver coil. This paper derives the relationship between the scaled factor dimensions and the coil properties. This relationship can be implemented during the manufacturing and design phase of the IWPT system, as the secondary coil can be modified by a scaling factor to shift the resonant frequency to match the primary coil which reduces computation time using FEM based software. This paper also presents the simulation and measurement results that show the eligibility of the Kapton-based RX coil as a wearable sensor and its performance superiority compared to the other state-of-the-art flexible and rigid coil-based WPT systems. In future works, the circuit components such as a rectifier, DC–DC converter, amplifier, and transmitter can be designed on a Complementary Metal-Oxide Semiconductor (CMOS) chip and integrated with the Kapton substrate for a miniaturized wearable sensor application. Another important aspect for a wearable sensor is the bending effect which will be included in the analytical modeling and validation for different bending scenarios in our future works.

## Figures and Tables

**Table 1:** Properties of the conductor used in the coil.

Conductor Material	Relative Permetivity	Bulk	Resistivity	Trace
		Conductivity	$\rho_{c}$	Thickness
		(S/m)	(Ω-m)	(μm)
Silver	0.99	61× $10^{6}$	1.64 × $10^{-8}$	0.25

**Table 2:** Geometrical characteristics of the coils.

Ratio	Outer	Inner	Conductor	Space
	Diameter	Diameter	Trace	Between
	$d_{o}$	$d_{i}$	Width	Traces
	(mm)	(mm)	*w* (mm)	*s*(mm)
1.00	10.00	2.00	1.00	0.50
1.20	12.00	2.40	1.20	0.60
1.35	13.50	2.70	1.35	0.67
1.65	16.50	3.30	1.65	0.82
1.80	18.00	3.60	1.80	0.90
1.95	19.50	3.90	1.95	0.97
2.00	20.00	4.00	2.00	1.00

**Table 3:** Comparison with the state-of-the-art works.

Criteria	[10]	[11]	[15]	This Work
RX Substrate type	Parylene film	Kapton	FR4	Kapton
RX conductor type	Gold	Silver	Copper	Silver
RX coil type	Dipole	Split-ring	Spiral	Spiral
RX coil dimension (mm${}^{2}$)	5 × 27	14 × 15	11.25 × 6	20 × 20
TX coil dimension (mm${}^{2}$)	NA	14 × 15	10.5 × 10.5	45 × 45
Frequency	825 MHz	2.4 GHz	7.15 MHz	405 MHz
Distance of operation (mm)	100	5	5	10
PTE	0.086%	0.79%	4.1%	42%
